# Film Coating of Small Molded Tablets for Pediatric Formulations with Rapid Disintegration and Bitterness-Masking Properties

**DOI:** 10.3390/molecules30102142

**Published:** 2025-05-13

**Authors:** Yuki Takahashi, Takayuki Furuishi, Etsuo Yonemochi

**Affiliations:** 1 Alfresa Pharma Corporation, 3038-2 Serada-cho, Ota City 370-0426, Gunma, Japan; 2 Juntendo University Faculty of Pharmacy, 6-8-1 Hinode, Urayasu City 279-0013, Chiba, Japan; 3 School of Pharmacy at Narita, International University of Health and Welfare, 4-3 Kozunomori, Narita City 286-8686, Chiba, Japan

**Keywords:** molded tablets, film coating, bitterness-masking, rapid disintegration, pediatric formulation

## Abstract

The bitter taste of drugs is an important impediment to medication adherence for pediatric patients. To develop a formulation that can be easily taken by pediatric patients, we prepared film-coated molded tablets to mask their bitterness and investigated their properties. We manufactured 5 and 3 mm film-coated tablets, which were easy for children to swallow. The tablets also exhibited rapid disintegration (≤30 s), making them suitable for tube administration. The formulation of the film-coating layer was experimentally optimized. Tablets (measuring 5 and 3 mm thick) containing the model drug dextromethorphan hydrobromide were film-coated by weight of the uncoated tablets (4% by weight). These tablets rapidly disintegrated and masked the bitterness for 10 s. An examination of the coated tablets revealed that the film covered the periphery, which may mask the bitterness. The findings demonstrate that coating small molded tablets with a film enables the manufacture of tablets that could be more tolerable for pediatric patients and suitable for tube administration.

## 1. Introduction

Pediatric formulations became mandatory in the United States in 2003 and in Europe in 2007 [1,2]. In contrast, Japan has no regulations mandating pediatric drug development. Between 2007 and 2015, the number of newly approved pediatric formulations in Europe and the United States was more than four times higher than in Japan [3], highlighting Japan’s lag in pediatric formulation development. To address this issue, the Act on Securing Quality, Efficacy, and Safety of Products Including Pharmaceuticals and Medical Devices was amended in Japan in 2019 to promote pediatric drug research and development. This change introduced a new designation system for “drugs for specific use” [4]. This system offers benefits for pediatric drug development, such as priority review for designated drugs, and aims to promote pediatric drug research and development. Consequently, pediatric formulation design has recently gained increased attention in Japan.

The bitter taste of drugs is a key factor influencing medication adherence, particularly in pediatric patients. Bitterness often leads to medication refusal, especially in pediatric patients, who have a low tolerance for bitter flavors [5]. Therefore, designing drugs without bitterness is crucial for improving medication adherence and ensuring effective treatment outcomes in pediatric patients.

Pharmaceutical syrups are commonly used pediatric formulations. However, some children find them unappealing due to their unpleasant smell and excessive sweetness [6]. The World Health Organization recommends oral solid formulations for pediatric drug therapy [7]. A survey on pediatric formulations revealed that mini-tablets are more popular than syrups [8]. Mini-tablets, typically 2–4 mm in diameter, are gaining attention as a suitable option for children. The survey also reported that children aged ≥3, ≥2, and ≥1 years can swallow tablets with diameters of 8 mm, 5 mm, and 4 mm, respectively [9,10], suggesting that smaller tablets are more easily taken by younger children.

In addition to their bitterness-masking ability and small size, pediatric formulations should also be suitable for tube administration. This is important because pediatric feeding tubes have a narrow inner diameter and are more susceptible to tube blockage [11]. Therefore, tablets composed primarily of water-soluble additives are preferred over those with large amounts of insoluble additives for tube administration. Additionally, using rapidly disintegrating tablets reduces the burden on healthcare workers and caregivers, as a longer disintegration time increases the preparation time before tube administration. These considerations suggest that tasteless, small, rapidly disintegrating tablets containing water-soluble additives are ideal for pediatric use. Such dosage forms can be produced by applying a film coating to small molded tablets.

Conventional tablets are produced by compressing a mixture of raw materials or granulated substances with a compression force range of 5–15 kN. In contrast, molded tablets are made by compressing wet powders at a much lower force range (100–300 N) and then drying the remaining solvent. Molded tablets have higher porosity levels compared to conventional tablets, which give them the characteristic of rapid disintegration [12]. Despite their porosity, these tablets are strong enough to undergo film coating. Typically, rapidly disintegrating tablets contain water-insoluble disintegrating agents such as low-substituted hydroxypropyl cellulose and crospovidone. However, molded tablet formulations do not contain any disintegrating agents; instead, they are composed entirely of water-soluble additives. This allows molded tablets to disintegrate rapidly and makes them suitable for tube administration.

Due to their rapid disintegration, molded tablets may dissolve in the oral cavity, exposing patients to the bitter taste of the active pharmaceutical ingredient (API). To make these tablets suitable for pediatric patients, bitterness masking is essential. One published method involves masking the bitterness of molded tablets by coating the API with a water-insoluble polymer and incorporating bitterness-masking granules [13]. However, the manufacturing process of molded tablets requires the kneading of bitterness-masking granules with a solvent. As a result, the choice of solvent must be limited to one that does not dissolve the masking layer. Film coatings, which do not limit the formulation of molded tablets, are considered a suitable approach for masking bitterness. The film coating acts as a physical barrier, preventing bitter drugs from coming into contact with the taste buds, allowing patients to take the tablets without experiencing a bitter taste. While film-coated molded tablets maybe slightly harder to swallow compared to uncoated molded tablets due to their lack of oral disintegration, smaller tablet sizes are generally easier for patients to swallow. Additionally, by leveraging their ability for rapid disintegration, film-coated molded tablets can be designed for tube administration. This rapid disintegration is also expected to reduce the risk of choking in pediatric patients.

**Figure 1:**
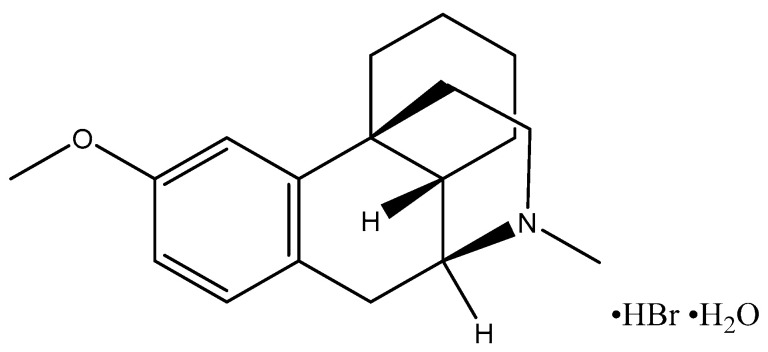
Chemical structure of dextromethorphan hydrobromide hydrate (DEX).

Based on these considerations and with the goal of developing an oral solid formulation for pediatric patients, this study aimed to design a film-coated formulation suitable for masking the bitterness of molded tablets. The bitterness-masking ability was evaluated using a taste-sensing device. As previously described, trehalose was used as a binder for the molded tablets [12]. Tablet diameters of 5 mm and 3 mm were examined, both suitable for pediatric use. The 5 mm size represents the smallest diameter of conventional tablets, while 3 mm corresponds to mini-tablets. Dextromethorphan hydrobromide hydrate (Dex; Figure 1), a strongly bitter, non-narcotic antitussive commonly used in pediatric medicine, was selected as the model drug. Effective bitterness masking of Dex is crucial in the development of pediatric formulations to improve patient compliance.

## 2. Results and Discussion

### 2.1. Optimization of Film Coating Formulation

Based on the formulation determined by the mixture design outlined in Table 1, placebo-molded tablets with a diameter of 5 mm were coated, and the results are presented in Table 2. The hardness and disintegration times were set for the response because hardness is considered important for tablet handling and the disintegration time for tube administration. Table 3 shows the hardness values of the film-coated tablets. The main effects of hypromellose (TC-5E), polyethylene glycol (PEG)-6000, and talcum (talc), along with the interaction between TC-5E and PEG-6000, significantly influenced the tablet hardness (*p* < 0.05). Specifically, the estimated regression coefficients for TC-5E and PEG-6000 were positive, indicating that the hardness increased as their mixing ratio increased. Conversely, the estimated regression coefficient for the interaction between TC-5E and PEG-6000 was negative, suggesting that the combination of these two components reduced the tablet hardness.

A previous study reported that using PEG as a plasticizer in hydroxypropyl methylcellulose (HPMC) films, along with increasing the PEG ratio, decreased the film’s tensile strength while increasing its elongation rate [14]. This, along with the findings from the present study, suggest that increasing the PEG ratio reduces the hardness of film-coated tablets due to PEG’s plasticizing effect. However, as shown in Table 4, only TC-5E had a statistically significant effect on the disintegration time (*p* < 0.05). The positive regression coefficient for TC-5E indicates that the disintegration time increased as the TC-5E ratio increased. These results demonstrate that the TC-5E mixing ratio represents a trade-off between achieving higher hardness and maintaining rapid disintegration.

**Figure 2:**
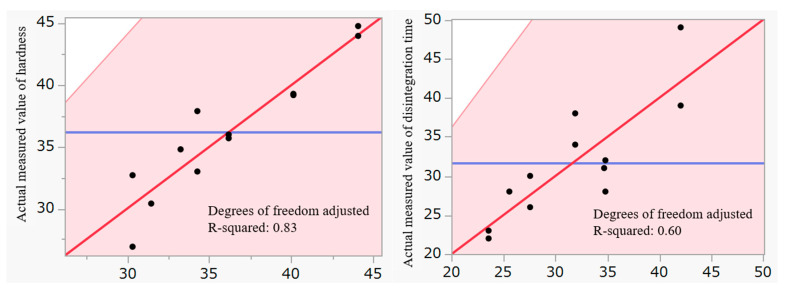
Plots of the predicted and actual measured values of hardness (**left**) and disintegration time (**right**).

Figure 2 presents plots of the predicted hardness and disintegration time values based on the model equation, along with their corresponding measured values. The adjusted R-squared values for the hardness and disintegration time models were 0.83 and 0.60, respectively, indicating a strong fit for the hardness model and a moderate fit for the disintegration time model. The lack-of-fit test did not reveal any significant issues with the model. Based on these findings, the model was considered appropriate.

**Figure 3:**
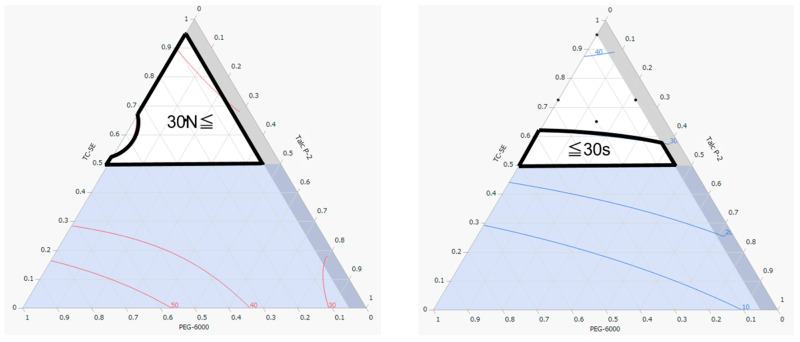
Design spaces for hardness (**left**) and disintegration time (**right**).

The design spaces for the hardness and disintegration times of the film-coated tablets are shown in Figure 3. For ease of handling, the film coating should result in higher hardness compared to uncoated tablets. In the hardness design space (Figure 3, left), the area circled in black indicates a range of ≥30 N, which exceeds the hardness of uncoated tablets (29 N). Additionally, because the objective was to produce film-coated tablets with rapid disintegration, the area circled in black in the disintegration designed space (Figure 3, right) represents disintegration times of ≤30 s, in line with the U.S. Food and Drug Administration’s recommendations for orally disintegrating tablets [15].

**Figure 4:**
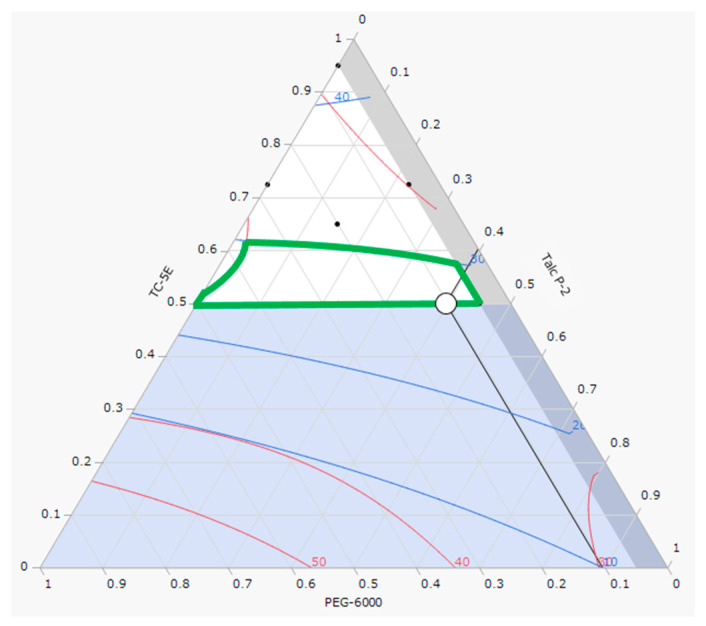
Design space where the ranges of hardness and disintegration time are superimposed. (Inside the green frame: hardness of ≥30 N and disintegration time of ≤30 s; 〇: optimal condition.)

Figure 4 illustrates the design space where the hardness (≥30 N) and disintegration time (≤30 s) (Figure 3) are overlaid. Using JMP version 17 statistical analysis software, we identified the optimal mixing ratios for each factor, maximizing the satisfaction function within the design space (〇 in Figure 4). This optimal formulation is detailed in Table 5. Film-coated tablets manufactured based on this formulation were predicted to have a hardness of 6 N and a disintegration time of 16 s, both of which exceeded those of uncoated tablets (Table 6).

### 2.2. Coating of 5 mg Dex Tablets

Molded tablets with a 5 mm diameter, containing 5 mg Dex as the model drug, were produced and coated with a film at an uncoated tablet mass ratio of 3%, based on the optimal formulation shown in Table 5. Although the uncoated tablets had low hardness (10.9 N), no breakage occurred during the coating process. The coated tablets exhibited a hardness that was 4.8 N higher than that of the uncoated tablets, and their disintegration time was 11 s longer (Table 7). These results fell within the 95% confidence intervals of the predicted values from the optimized coating formulation (Table 6), indicating the model’s high prediction accuracy for hardness and disintegration time.

**Figure 5:**
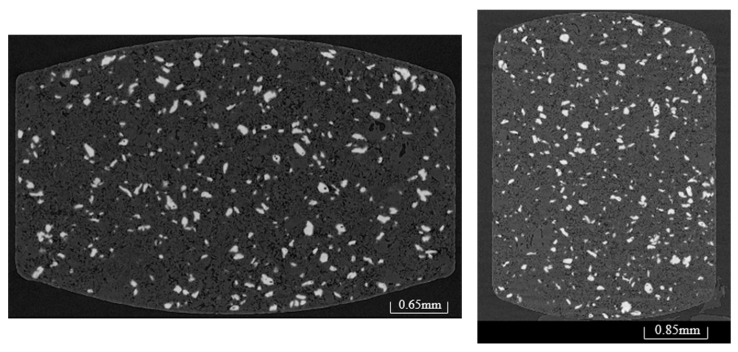
X-ray CT images of the inside of Dex 5 mg coated tablets (**left**) and Dex 2.5 mg coated tablets (**right**). The white dots indicate DEX.

**Figure 6:**
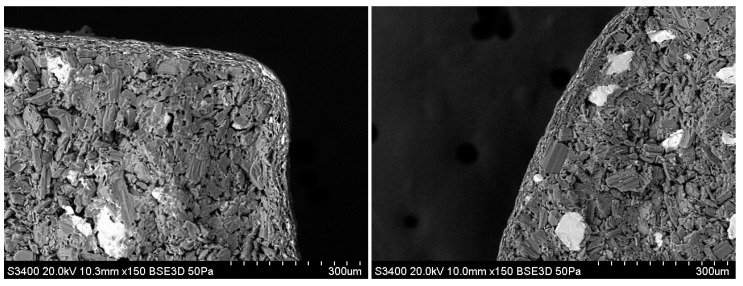
Scanning electron microscopy images of the film-coating layer of 5 mg Dex coated tablets (**left**) and 2.5 mg Dex coated tablets (**right**) (150× magnification).

The molded tablets were composed of water-soluble additives, which may have undergone plastic deformation due to moisture during the film-coating process. However, X-ray computed tomography (CT) images of the coated tablets’ interiors confirmed that the molded tablets were successfully coated with a film without undergoing plastic deformation (Figure 5). Additionally, while conventional tablets have a porosity of 9.2% [12], the coated molded tablets in this study exhibited more than double the porosity at 24.7%. The findings confirmed that the film-coated tablets had the high-porosity characteristics of the molded tablets. In addition, the inside of the film-coated tablets was observed using electron microscopy because the periphery of the uncoated tablets must be covered with a film layer to mask the bitter taste. This examination confirmed that the periphery of the coated tablets was effectively covered with a film layer (Figure 6).

### 2.3. Bitterness of 5 mg Dex-Coated Tablets

**Figure 7:**
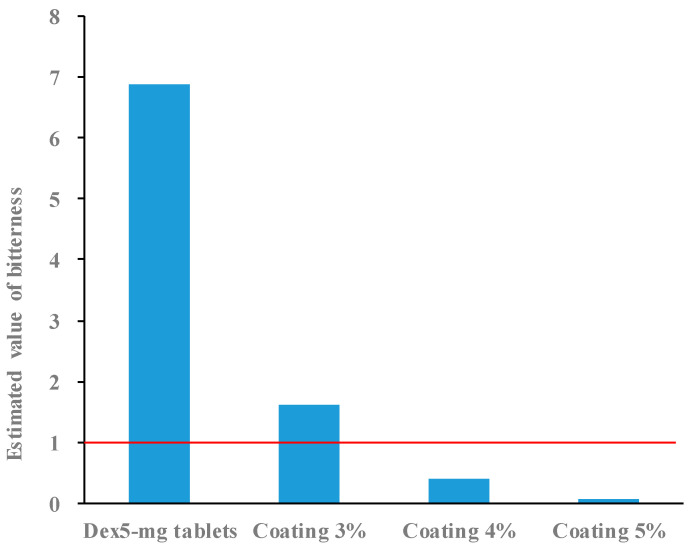
Estimated bitterness values of tablets 5 s after being placed in the test solution (*n* = 1). Tablets with values below the red line were considered free of bitterness.

**Figure 8:**
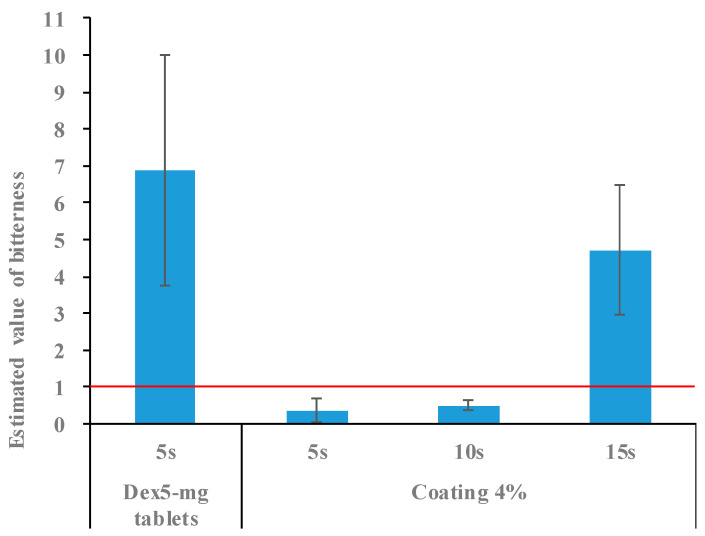
Estimated bitterness values of tablets for up to 15 s after being placed in the test solution (*n* = 3). Tablets with a value below the red line were considered free of bitterness.

The Dex tablets were coated with film mass ratios of 3%, 4%, and 5%. The bitterness-masking effect of these tablets was evaluated for 5 s using a taste sensor. The coated Dex tablets had an estimated bitterness value of 6.9 after 5 s in the test solution. Additionally, the bitterness value decreased in the order of tablets coated with mass ratios of 3%, 4%, and 5% (Figure 7), indicating that the masking effect improved as the coating ratio increased. However, the tablets with a 3% coating mass ratio did not meet the threshold of an “estimated bitterness value of <1”, which corresponds to the absence of a bitter taste for patients. These results indicate that a coating layer with a mass ratio of at least 4% is necessary to effectively mask bitterness. Conversely, increasing the coating ratio not only enhanced the bitterness-masking effect but also extended the disintegration time. Tablets coated with a 5% mass ratio had a disintegration time of 33 s, exceeding the target of ≤30 s. Based on these results, a coating ratio of 4% provided a balance between effective bitterness masking (5 s) and an acceptable disintegration time of 25 s. We then evaluated the bitterness-masking effect for up to 15 s using tablets with a 4% coating mass ratio. The estimated bitterness value of these tablets remained <1 (0.5) for up to 10 s after immersion in the test solution but increased to >1 (4.7) at 15 s (Figure 8). Therefore, a 4% film coating was effective at masking the bitterness for 10 s. Since small tablets can be easily taken, we believe that pediatric patients can swallow them without experiencing any bitterness if it is masked for at least 10 s.

### 2.4. Bitterness of 2.5 mg Dex Coated Tablets

Since the bitterness of 5 mg Dex tablets with a 5 mm diameter could be masked by applying a film coating with a mass ratio of 4%, we next examined whether we could similarly mask the bitterness of 3 mm diameter molded tablets containing 2.5 mg of Dex. Table 8 shows the physical properties of the uncoated and coated 2.5 mg Dex tablets. A film coating with a mass ratio of 4% increased the hardness by 6.9 N compared with that of the uncoated tablets. Although the disintegration time of the coated tablets increased by 16 s, it remained under 30 s. Similar to the 5 mg Dex coated tablets, X-ray CT examination of the inside of the coated tablets confirmed that the molded tablets were coated with a film without plastic deformation (Figure 5). The coated tablets exhibited high porosity (32.8%). Electron microscopy revealed that the periphery of the molded tablets was covered with a film coating (Figure 6).

**Figure 9:**
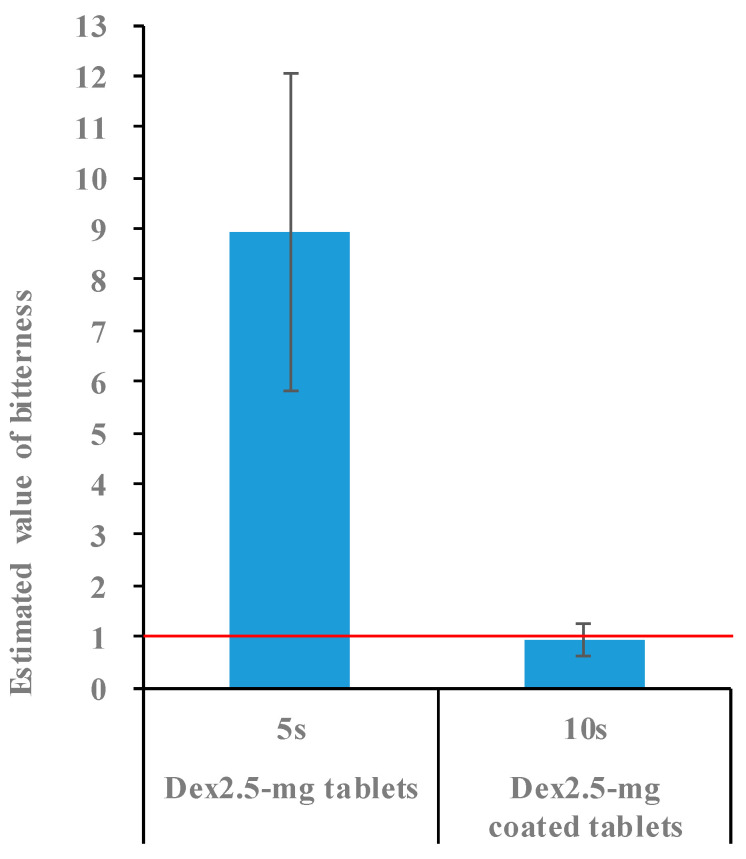
Estimated bitterness values of uncoated and coated 2.5 mg Dex tablets (*n* = 3). Tablets with values below the red line were considered free of bitterness.

To measure the bitterness, the Dex content was set to 5 mg. Two uncoated or coated 2.5 mg Dex tablets were used for each measurement. Figure 9 shows an evaluation of the estimated bitterness values. While the uncoated 2.5 mg Dex tablets had an estimated bitterness value of 8.9 after being soaked in the test solution for 5 s, the estimated bitterness value of the coated 2.5 mg Dex tablets was <1 (0.9), even after 10 s, indicating effective bitterness masking. Based on these findings, we successfully manufactured coated 2.5 mg Dex tablets with rapid disintegration (≤30 s) that effectively masked the bitterness for 10 s after being placed in the oral cavity.

## 3. Materials and Methods

### 3.1. Materials

Pearlitol 25C (Roquette Japan, Tokyo, Japan), trehalose (Hayashibara Co., Ltd., Okayama, Japan), and 99% ethanol (Japan Alcohol Trading Co., Ltd., Tokyo, Japan) were used. The Dex was purchased from Alps Pharmaceutical, Inc. Co., Ltd. (Gifu, Japan). The coating base for the film-coating layer was TC-5E, a low-viscosity (3 mPa s) HPMC product purchased from Shin-Etsu Chemical Co., Ltd. (Tokyo, Japan). Macrogol 6000 plasticizer (PEG-6000) was purchased from NOF Corporation (Tokyo, Japan). Talc P-2 lubricant was purchased from Matsumura Sangyo Co., Ltd. (Osaka, Japan). Purified water was used in all experiments.

### 3.2. Mixture Design for Film-Coating Formulation

The formulation of the film-coating layer was optimized using a mixture design to produce film-coated tablets with greater hardness than that of uncoated tablets while maintaining a disintegration time of less than 30 s. In this design, the sum of all factors was maintained at 100%. JMP version 17 statistical analysis software (SAS Institute, Cary, NC, USA) was utilized to create the mixture design and analyze the responses. The mixture design was based on the D optimization criteria.

**Figure 10:**
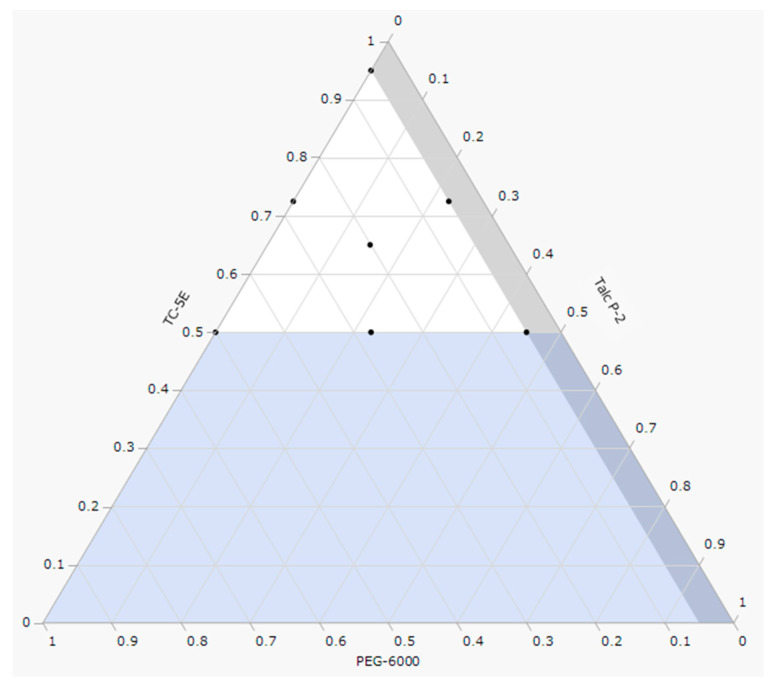
Experimental points of the mixture design (black dots) and the range of formulations examined (white area).

The selected factors were TC-5E, PEG-6000, and Talc P-2 (all %). The evaluated responses included the tablet hardness (N) and disintegration time (s). Table 9 displays the range of mixing ratios for each factor, while Table 1 outlines the film-coating layer formulations determined by the mixture design for the 12 batches. To estimate the experimental error, 5 of the 12 conditions were repeated (conditions 1 and 9, 2 and 12, 3 and 5, 7 and 10, 8 and 11 were the same). Figure 10 illustrates the experimental points of the mixture design (black dots) and the range of formulations investigated (white areas).

### 3.3. Statistical Analysis of Responses

The responses obtained from the mixture design (Table 1) are presented in Table 2. The responses were fitted to a model derived using the least-squares method, and the effects of the factors on the responses were analyzed using JMP version 17 statistical analysis software. Factors with *p*-values ≥ 0.2 were excluded from the model through pooling. Statistical significance was set at *p* < 0.05.

### 3.4. Optimization of the Film-Coating Formulation

A satisfaction function was employed to determine the optimal factor values. The optimal value for each factor was identified as the point where the overall satisfaction function reached its maximum [16]. The overall satisfaction function was defined as the geometric mean of the satisfaction function for multiple responses, with a scale ranging from 0 to 1.

### 3.5. Preparation of Molded Tablets

The batch size for the molded tablets was set at 30 g. Placebo tablets were prepared following the formulation shown in Table 10. Pearlitol 25C diluent and trehalose binder were first placed in a model R-8 mill (Nippon Rikagaku Kikai, Tokyo, Japan) and mixed for 10 s. Subsequently, an ethanol and water solvent solution was added to the mixture, and the blend was kneaded for 30 s to form a wet powder. For each tablet, 60 mg of the wet powder was added to fill a 5 mm mortar, followed by compression at a molding pressure of 300 N. This was done using a model RTG-1210 Tensilon universal testing machine (A&D, Tokyo, Japan) with a 5 mm diameter pestle and an R of 6.5. The compressed material was then dried to produce the placebo molded tablets.

To prepare tablets containing the model drug, a portion of the Pearlitol 25C was replaced with Dex, and the wet powder was prepared in the same manner as described for the placebo tablets. The formulations for the 5 mg and 2.5 mg Dex tablets are provided in Table 10. The Dex content was determined based on a single dose for children aged 3 months to 7 years, by dividing the maximum daily dose of 20 mg into four oral doses. Tablets containing 5 mg of Dex were prepared by compressing 60 mg of wet powder (solid content) at 150 N, which was expected to achieve a disintegration time of ≤30 s, using the same mortar and pestle as described for the placebo tablets. After compression, the tablets were dried to produce 5 mg Dex tablets.

Dex tablets (2.5 mg) were prepared using half the mass of the Dex 5 mg tablets (30 mg), with a 3 mm mortar and pestle. The molding pressure for the 2.5 mg Dex tablets was set at 120 N, which was expected to result in a disintegration time of ≤30 s. All other conditions remained the same as those for the 5 mg Dex tablets.

### 3.6. Film Coating of Molded Tablets

To optimize the film coating, 12 batches were coated using a model HC-LABO tablet coating device (Freund Corporation, Japan) according to the mixture design formulation outlined in Table 1. The film coating was carried out by mixing 30 placebo tablets (produced as described earlier) with 2000 general conventional tablets (6 mm in diameter, 100 mg mass). The uncoated tablets were used with a mass ratio of 3% to determine the mass of the coating layers. The coating solution was prepared by dissolving TC-5E, PEG-6000, and Talc P-2 in purified water and stirring for at least 40 min, yielding a solid concentration of 10 wt%. The specific film-coating conditions are detailed in Table 11.

Film-coated 5 mg Dex tablets were prepared in the same manner as described above, following the formulation provided in Table 5. To evaluate the bitterness-masking effect based on the mass of the film-coating layer, three coating masses were prepared, corresponding to uncoated tablet mass ratios of 3%, 4%, and 5%.

**Figure 11:**
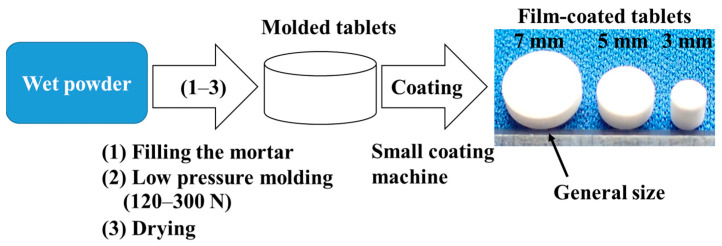
Manufacturing flow for production of film-coated tablets.

Film-coated 2.5 mg Dex tablets were prepared based on the formulation described in Table 5 for 5 mg Dex tablets. In addition, the film coating of 2.5 mg Dex tablets was performed by mixing 40 2.5 mg Dex tablets with 2000 general conventional tablets (with a diameter of 6 mm and a mass of 100 mg), resulting in film-coated tablets with an uncoated tablet mass ratio of 4% (2.5 mg Dex coated tablets). Forty film-coated tablets were considered necessary for evaluation. Figure 11 depicts the manufacturing process for the film coating of the molded tablets.

### 3.7. Tablet Hardness

The hardness of 10 tablets was measured using a KHT-40N hardness tester (FUJIWARA SCIENTIFIC Co., Ltd., Tokyo, Japan). The mean values were calculated and are presented here.

### 3.8. Disintegration Time

Purified water at 37 ± 2 °C was used as the test solvent. The disintegration time of the tablets was measured using a model NT-210 disintegration tester (Toyama Sangyo, Osaka, Japan) according to the disintegration test method described in the 18th Revised Japanese Pharmacopoeia. The mean disintegration time of six tablets was determined. An auxiliary board was used because the film-coated tablets floated during the test. An auxiliary cylinder was used to measure the tablets with a diameter of 3 mm owing to their small size.

### 3.9. Bitterness Evaluation of Coated Tablets

Each sample was placed in a container containing aqueous 10 mM KCl as the diluent and immersed for a specified time period. The diluent was filtered through a 75 µm mesh sieve, and the filtrate was subjected to bitterness measurement using a TS-5000Z taste-sensing device (Intelligent Sensor Technology, Kanagawa, Japan) equipped with a BT0 sensor. The bitter taste sensor mimics the mechanism by which the human tongue perceives bitter tastes. The sensor measures the change in membrane potential that occurs when a taste substance is adsorbed onto an artificial lipid membrane and quantifies the intensity of the bitterness [17]. Each sample was measured four times, and the estimated bitterness value was calculated using data from three measurements, excluding the first. The estimated bitterness value was determined by multiplying the sensor value by a specific coefficient (0.3 for BT0 sensor). Differences in taste were considered distinguishable with a value difference of ≥1 [18]. Tablets with an estimated bitterness value < 1 were judged to have no bitterness (no difference from the blank solution) in this study. For each measurement, one 5 mg Dex coated tablet or two 2.5 mg Dex coated tablets were used, ensuring a total Dex content of 5 mg.

### 3.10. X-Ray CT

An X-ray CT system for high-resolution measurements (v|tome|x m 240/180; GE Sensing and Inspection Technologies, Tokyo, Japan) was used to examine the internal structures of the tablets. The tube voltage was set at 100 kV and the current at 50 µA. VG Studio MAX 3.2 (Volume Graphics, Nagoya, Japan) was used for the image analysis.

### 3.11. Scanning Electron Microscopy

The tablets were split in the diametric direction and the split surface was observed at 150× magnification using a model S-3400N scanning electron microscope (Hitachi High Technologies, Tokyo, Japan).

## 4. Conclusions

To develop a formulation suitable for pediatric patients, this study investigated the film coating of small molded tablets. The formulation of the film-coating layer was optimized using a mixture design. The optimal film-coating layer-to-uncoated tablet mass ratio was evaluated at three levels (3%, 4%, and 5%) using 5 mg Dex tablets with a 5 mm diameter. At a 4% coating ratio, the bitterness of the tablets was masked for 10 s and the tablets disintegrated (≤30 s). Similarly, when 2.5 mg Dex tablets (3 mm in diameter) were coated at a 4% mass ratio, the bitterness masking and rapid disintegration results were comparable to those of the coated 5 mg Dex tablets. Examinations of the interiors of both the 5 mg and 2.5 mg Dex coated tablets showed that the periphery of the molded tablets was covered with a film-coating layer, effectively masking the bitter taste of the API. These findings suggest that coating small molded tablets with a 4% film layer produces rapidly disintegrating coated tablets that are suitable for tube administration, and are also ideal for pediatric patients, as they do not exhibit bitterness for up to 10 s.

## Figures and Tables

**Table 1:** Formulation of the film-coating layer determined by the mixture design.

No.	Factor			
	TC-5E (%) (X1)	PEG-6000 (%) (X2)	Talc P-2 (%) (X3)	Repeat
1	50.0	5.0	45.0	—
2	72.5	5.0	22.5	—
3	95.0	5.0	0.0	—
4	72.5	27.5	0.0	—
5	95.0	5.0	0.0	No. 3
6	50.0	27.5	22.5	—
7	50.0	50.0	0.0	—
8	65.0	20.1	14.9	—
9	50.0	5.0	45.0	No. 1
10	50.0	50.0	0.0	No. 7
11	65.0	20.1	14.9	No. 8
12	72.5	5.0	22.5	No. 2

**Table 2:** Responses obtained from the mixture design.

No.	Response	
	Hardness, N (Y_1_)	Disintegration Time, s (Y_2_)
1	36.0	26
2	39.2	28
3	44.8	39
4	30.4	31
5	44.0	49
6	34.8	28
7	26.9	23
8	37.9	34
9	35.7	30
10	32.7	22
11	33.0	28
12	39.3	32

**Table 3:** Factors affecting hardness.

Item	Estimated Value (N)	Standard Error (N)	t-Value	*p*-Value
TC-5E	44.086588	1.40353	31.41	<0.0001 *
PEG-6000	68.931864	21.16757	3.26	0.0116 *
Talc P-2	27.402006	3.451996	7.94	<0.0001 *
TC-5E × PEG-6000	−102.4309	38.54277	−2.66	0.0289 *

* Statistically significant difference (*p* < 0.05).

**Table 4:** Factors affecting the disintegration time.

Item	Estimated Value (s)	Standard Error (s)	t-Value	*p*-Value
TC-5E	42.047464	3.088938	13.61	<0.0001 *
PEG-6000	−14.56181	46.58634	−0.31	0.762
Talc P-2	11.464492	7.597276	1.51	0.1697
TC-5E × PEG-6000	33.429604	84.82631	0.39	0.7038

* Statistically significant difference (*p* < 0.05).

**Table 5:** Optimal formulation for film-coating layer.

Mixing Purpose	Raw Material	Ratio (wt%)
Coating Base	TC-5E	50.0
Plasticizer	PEG-6000	10.3
Lubricant	Talc P-2	39.7
Solvent	Purified water	(a)
Total (excluding solvent)		100

(a) The solid content concentration of the coating solution was 10 wt%.

**Table 6:** Expected increase after coating based on optimal formulation.

	Response	
	Hardness, N	Disintegration Time, s
Predicted values for film-coated tablets (①)	35 (33–38)	27 (21–33)
Actual results for uncoated tablets (②)	29	11
Expected increase after coating (①–②)	6 (4–8)	16 (10–22)

Numbers in parentheses indicate 95% confidence intervals.

**Table 7:** Responses of uncoated and coated 5 mg Dex tablets.

	Response	
	Hardness, N	Disintegration Time, s
Uncoated tablets (①)	10.9	12
Coated tablets (②)	15.7	23
Difference (②–①)	4.8	11

**Table 8:** Physical properties of uncoated and coated 2.5 mg Dex tablets.

	Response	
	Hardness, N	Disintegration Time, s
Uncoated tablets (①)	16.0	13
Coated tablets (②)	22.9	29
Difference (②–①)	6.9	16

**Table 9:** Range of mixing ratio of each factor.

Factor	Range	
	Lower Limit (%)	Upper Limit (%)
TC-5E (X1)	50	95
PEG-6000 (X2)	5	50
Talc P-2 (X3)	0	45

**Table 10:** Formulation of the molded tablets used in this study.

Mixing Purpose	Raw Material	Ratio (wt%)	
		Placebo Tablets	5 or 2.5 mg Dex Tablets
Diluent	Pearlitol 25C	89.6	81.3
Binder	Trehalose P	10.4	10.4
Active component	Dex	—	8.3
Solvent	99% ethanol	5.0 ^(a)^	5.0 ^(a)^
	Purified water	7.1 ^(a)^	7.1 ^(a)^
Total (excluding solvent)		100	100

^(a)^ Ratio to solid content.

**Table 11:** Film-coating conditions.

Task	Setting Item	Setting Value
Preheating	Supply air temperature (°C)	65
	Supply air volume (m^3^/min)	0.4
	Static pressure inside pan	−10
	Pan rotation speed (rpm)	5
	Exhaust temperature (°C)	45
Spraying	Supply air temperature (°C)	65
	Supply air volume (m^3^/min)	0.4
	Static pressure inside pan	−10
	Pan rotation speed (rpm)	25
	Liquid velocity (g/min)	1.2
	Spray flow meter (NL/min)	40
	Spray pressure (mPa s)	0.12
	Exhaust temperature (°C, approximate)	≥43 °C
Drying	Supply air temperature (°C)	65
	Supply air volume (m^3^/min)	0.4
	Static pressure inside pan	−10
	Pan rotation speed (rpm)	5
	Exhaust temperature (°C)	47
Cooling	Supply air temperature (°C)	OFF
	Supply air volume (m^3^/min)	0.4
	Static pressure inside pan	−10
	Pan rotation speed (rpm)	5
	Exhaust temperature (°C)	≤40 °C

## Data Availability

Data will be made available on request.

## Abbreviations

The following abbreviations are used in this manuscript:

API	Active pharmaceutical ingredient
Dex	Dextromethorphan hydrobromide hydrate
HPMC	Hydroxypropyl methylcellulose
